# The Problem of Limited Inter-rater Agreement in Modelling Music Similarity

**DOI:** 10.1080/09298215.2016.1200631

**Published:** 2016-07-05

**Authors:** Arthur Flexer, Thomas Grill

**Affiliations:** ^a^ Austrian Research Institute for Artificial Intelligence (OFAI), Intelligent Music Processing and Machine Learning Group, Vienna, Austria.

**Keywords:** information retrieval, perception, evaluation

## Abstract

One of the central goals of Music Information Retrieval (MIR) is the quantification of similarity between or within pieces of music. These quantitative relations should mirror the human perception of music similarity, which is however highly subjective with low inter-rater agreement. Unfortunately this principal problem has been given little attention in MIR so far. Since it is not meaningful to have computational models that go beyond the level of human agreement, these levels of inter-rater agreement present a natural upper bound for any algorithmic approach. We will illustrate this fundamental problem in the evaluation of MIR systems using results from two typical application scenarios: (i) modelling of music similarity between pieces of music; (ii) music structure analysis within pieces of music. For both applications, we derive upper bounds of performance which are due to the limited inter-rater agreement. We compare these upper bounds to the performance of state-of-the-art MIR systems and show how the upper bounds prevent further progress in developing better MIR systems.

## 1. Introduction

Probably the most important concept in Music Information Retrieval (MIR) is that of *music similarity*. Proper modelling of music similarity is at the heart of every application allowing automatic organization and processing of music databases. Music similarity can be modelled at many different levels, e.g. between complete pieces of music or by exploring structure within pieces of music. Respective tasks at the annual ‘Music Information Retrieval Evaluation eXchange’ (MIREX^1^, 2006; Downie et al. , 2014) are the ‘Audio Music Similarity and Retrieval’ (AMS) task and the ‘Music Structural Segmentation’ (MSS) task. MIREX is an annual evaluation campaign for MIR algorithms allowing for a fair comparison in standardized settings in a range of different tasks. As such it has been of great value for the MIR community and an important driving force of research and progress within the community. It has even been stated that evaluation campaigns like MIREX ‘define de facto the topics that new contributors to the MIR field will work on’ (Serra et al. , 2013, p. 33).

Only some of the MIREX tasks directly involve human subjects that actually evaluate the results. Examples are ‘symbolic melody similarity’ and the AMS task where human graders evaluate pairs of query/candidate songs computed by participating algorithms. The human graders are asked to rate whether these query/candidate pairs ‘sound similar’ (AMS task) or to ‘evaluate the melodic similarity of two works’ (Jones, Downie, & Ehmann 2007) (symbolic melody similarity task). The majority of the tasks are not ‘user-centric’ but what has been termed ‘systems-based’ (Schedl, Flexer, & Urbano 2013), i.e. evaluation of algorithms on digital data bases consisting of music and annotations. Examples include audio genre or tag classification, audio onset detection or the MSS task. In all these systems-based tasks a form of human annotation (genres, onsets, segmentations, etc.) is treated as ‘ground-truth’, which is used to evaluate the performance of all participating algorithms. Specifically, in the MSS task, the participating algorithms produce structural segmentations (structural boundaries and labels denoting repeated segments) of music pieces which are part of large standardized collections comprising several hundred items. The music pieces are generally kept anonymous, but have each been annotated by one or more human listeners.

No matter if the evaluation is user-centred or systems-based, the quantitative relations modelled via algorithms should mirror the human perception of music. However, human perception of music is highly subjective with potentially low inter-rater agreement. This means, e.g. that if different human subjects are asked to rate the same song pairs according to their perceived similarity, only a certain amount of agreement can be expected due to a range of subjective factors. The same holds for annotation of music where different human subjects will not always agree on the correctness of a genre label or other semantic tag, or on the exact position of an onset or segment boundary. It seems to be evident that these levels of inter-rater agreement present a natural upper bound for any algorithmic approach, since it is not meaningful to have computational models that go beyond the level of human agreement.

This problem has not been given much attention in MIR research so far, and we therefore present a meta analysis of one task that directly involves human subjects in evaluation (MIREX AMS) and one task whose evaluation is based on human annotated labels (MIREX MSS). Our analysis of all MIREX AMS tasks from 2006 to 2014^2^ and of all MIREX MSS tasks from 2012 to 2015 will demonstrate that: (i) there is a low inter-rater agreement for both AMS and MSS; (ii) as a consequence there exists an upper bound of performance that cannot be surpassed by algorithmic approaches to AMS and MSS; (iii) this upper bound has very likely already been achieved years ago for AMS and not surpassed since then; the upper bound for MSS is already within reach for music from some specific genres.

In what follows we first review related work in Section 2, present the data for AMS and MSS in Section 3, give our results in Section 4, provide a discussion of results and make recommendations on how to improve future work on evaluating audio music similarity and structural segmentation in Section 5 and conclude in Section 6. Parts of the meta analysis of the AMS task have already been published at a conference (Flexer , 2014).

## 2. Related work

Our survey of related work will be given in three parts. First we review work that is concerned with the subjective nature of human perception of music similarity in general. Then we review work that specifically deals with the MIREX audio music similarity (AMS) and music structural segmentation (MSS) tasks.

### 2.1. Related work on subjective nature of music similarity

In discussing related work on the subjective nature of music similarity, we first focus on the way music similarity is defined and evaluated in the AMS task. The essence of the AMS task is to have human graders evaluate pairs of query/candidate songs. The query songs are randomly chosen from a test database and the candidate songs are recommendations automatically computed by participating algorithms. The human graders rate whether these query/candidate pairs ‘sound similar’ using both a BROAD (‘not similar’, ‘somewhat similar’, ‘very similar’) and a FINE score (from 0 to 10 or from 0 to 100, depending on the year the AMS task was held, indicating degrees of similarity ranging from failure to perfection). This very general notion of ‘sounding similar’ is one of the central points of criticism in this paper. A recent survey article on the ‘neglected user in music information retrieval research’ (Schedl et al. , 2013) has made the important argument that users apply very different, individual notions of similarity when assessing the output of music retrieval systems. It seems evident that music similarity is a multi-dimensional notion including timbre, melody, harmony, tempo, rhythm, lyrics, mood, etc. Nevertheless most studies exploring music similarity within the field of MIR, which are actually using human listening tests, are restricted to overall similarity judgments (see, e.g. Novello, McKinney, & Kohlrausch 2006; Pampalk , [p. 82]Pampalk:2006) thereby potentially blurring the many important dimensions of musical expression. There is very little work on what actually are important dimensions for humans when judging music similarity (see, e.g. Vignoli , 2004). Nevertheless some authors have developed context-based specifications of music similarity where users can interactively change the weighting of dimensions of similarity (e.g. Allan, Müllensiefen, & Wiggins , 2007; Pampalk, Dixon, & Widmer , 2004).

As to inter-rater agreement concerning annotations of music, a number of studies on music genre exist. As part of an early study (Lippens, Martens, Mulder, & Tzanetakis , 2004), 27 human subjects achieved a classification rate of 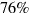 on a collection of 160 songs from six possible genres by listening to 30 s excerpts. Another study (Gjerdingen & Perrott , 2008) does not only report that human subjects are able to categorize music into genres given excerpts as short as a quarter second, but also that these subjects agree only in 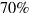 with the genre categorization as provided by music companies. In a study (Seyerlehner, Widmer, & Knees , 2010) comparing automatic to human based genre classification, it was shown that the performance of humans classifying songs into 19 genres, defined by the performing artists themselves, ranges from modest 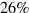 to 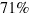 depending on the test subject. On average, automatic classifiers still performed ten percentage points worse. A study on grounding of everyday musical terms using acoustic properties of the corresponding music signals showed the same problematic results, prompting the author to raise the question (Aucouturier , 2009): ‘What if the current pattern recognition models were perfect? What if 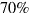 were in effect all that there is out there to find ...’.

Presenting even more fundamental criticism, the argument has been made (Wiggins , 2009) that music itself does not exist without human listening, without the psycho physiological effect of a stimulus on humans. Therefore no such thing as an immovable ‘ground’ exists in the context of music, which itself is subjective, highly context-dependent and not constant. Consequently something like a ground truth does not exist, since music is a cultural construct that has to be understood as the result of the collective action of many listeners. A similar conclusion has been drawn in a study on the feasibility of automatically annotating acousmatic music (Klien, Grill, & Flexer , 2012), where it is made clear that annotations of acousmatic and traditional music ‘need to be seen as communal, cultural constructs in their social context rather than objective “ground truths”.’

### 2.2. Related work on music similarity between music

In our review on related work we focus on papers directly discussing results of the AMS task, thereby addressing the problem of evaluation of audio music similarity.

After the first implementation of the AMS task in 2006, a meta evaluation of what had been achieved was published (Jones et al. , 2007). Contrary to all subsequent editions of the AMS task, in 2006 each query/candidate pair was evaluated by three different human graders. Most of the study was concerned with the inter-rater agreement of the BROAD scores of the AMS task as well as the ‘Symbolic Melodic Similarity (SMS)’ task, which followed the same evaluation protocol. To access the amount of agreement, the authors used Fleiss’ Kappa (Fleiss , 1971) which ranges between 0 (no agreement) and 1 (perfect agreement). Raters in the AMS task achieved a Kappa of 0.21 for the BROAD task, which can be seen as a ‘fair’ level of agreement. Such a ‘fair’ level of agreement (Landis & Koch , 1977) is given if the Kappa result is between 0.21 and 0.40, therefore positioning the BROAD result at the very low end of the range. Agreement in SMS is higher (Kappa of 0.37), which is attributed to the fact that the AMS task is ‘less well-defined’ since graders are only informed that ‘works should sound similar’ (Jones et al. , 2007). The authors also noted that the FINE scores for query/candidate pairs, which were judged as ‘somewhat similar’, showed more variance than the one judged as ‘very’ or ‘not’ similar. One of the recommendations of the authors was that ‘evaluating more queries and more candidates per query would more greatly benefit algorithm developers’ (Jones et al. , 2007), but also that a similar analysis of the FINE scores was also necessary.

For the AMS task 2006, the distribution of differences between FINE scores of raters judging the same query/candidate pair has already been analysed (Schedl et al. , 2013). For over 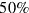, the difference between rater FINE scores is greater than 20. The authors also noted that this is very problematic since the difference between the best and worst AMS 2012 systems in terms of their average FINE scores was just 17.

In yet another analysis of the AMS task 2006, it has been reported (West , 2008) that a range of so-called ‘objective’ measures of audio similarity are highly correlated with subjective ratings by human graders. These objective measures are based on genre information, which can be used to automatically rank different algorithms producing lists of supposedly similar songs. If the genre information of the query and candidate songs are the same, a high degree of audio similarity is achieved since songs within a genre are supposed to be more similar than songs from different genres. It has therefore been argued that, at least for large-scale evaluations, these objective measures can replace human evaluation (West , 2008). However, this is still a matter of controversy within the music information retrieval community, see, e.g. Sturm (2013) for a recent and very outspoken criticism of this position.

A meta study of the 2011 AMS task explored the connection between statistical significance of reported results and how this relates to actual user satisfaction in a more realistic music recommendation setting (Urbano, Downie, McFee, & Schedl , 2012). The authors made the fundamental clarification that the fact of observing statistically significant differences is not sufficient. More important is whether this difference is noticeable and important to actual users interacting with the systems. Whereas a statistically significant difference can always be achieved by enlarging the sample size (i.e. the number of query/candidate pairs), the observed difference can nevertheless be so small that it is of no importance to users. Through a crowd-sourced user evaluation, the authors were able to show that there exists an upper bound of user satisfaction with music recommendation systems of about 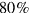. More concretely, in their user evaluation the highest percentage of users agreeing that two systems ‘are equally good’ never exceeded 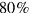. This upper bound cannot be surpassed since there will always be users that disagree concerning the quality of music recommendations. In addition, the authors were able to demonstrate that differences in FINE scores, which are statistically significant, are so small that they make no practical difference for users.

### 2.3. Related work on music similarity within music

The MIREX MSS task was introduced in 2009, initially employing evaluation data sets with a strong bias towards Western popular music. The first data set, termed ‘MIREX2009’, consists of songs by the Beatles with additional material from another smaller data set^3^. The 12 Beatles albums have been annotated in two versions, commonly denoted Beatles-TUT^4^ and Beatles-ISO^5^. Both sets of annotations reference the work of Alan W. Pollack^6^, but it is unclear if the same explicit guidelines were used for editing the original annotations in the context of Beatles-TUT and Beatles-ISO. The second data set (‘MIREX2010’) is equivalent to RWC-POP, the popular music subset of the RWC database^7^ (Goto , 2006). It contains annotations for Japanese and Western popular music. As described in Goto (2004), the songs of the RWC-POP subset (100 songs) were originally produced for the database. It is unclear whether the pieces have been post-annotated by human listeners, or if the annotations are derived from the underlying compositions. For the same RWC-POP data set, an alternative set of annotations^8^ has been produced in the scope of the QUAERO project^9^, using guidelines detailed in Bimbot, Deruty, Sargent, and Vincent (2012).

In an analysis of structural boundaries of Western-style popular music, the musical cues responsible for their perception have been examined (Bruderer, McKinney, & Kohlrausch , 2006). The study shows that the perception is not binary in nature, in the sense that a boundary exists or does not. On the contrary, the authors find a wide range of salience across different boundaries. Accordingly, ‘algorithms for automatic segmentation that intend to extract perceptually relevant structural elements should account for this range of salience in structural boundaries’.

In a survey article (Paulus, Müller, & Klapuri , 2010), the authors commented on the topic of MSS evaluation that ‘perhaps one of the largest problems in music structure analysis is not directly technical, but more conceptual: the ground truth for this task should be better defined. The need for this is indicated by the fact that the annotations made by two persons disagree to a certain degree. [...] The current results suggest that the structure description should not only be on a single level, but include also the information of hierarchical recurrences-similar to human perception’.

Subsequently, an improved, larger and stylistically more varied data set (termed ‘MRX10V2’ in the paper) has been discussed (Ehmann, Bay, Downie, Fujinaga, & De Roure , 2011) in comparison to the previously used MIREX data sets. This data set has later been adopted by MIREX under the name ‘MIREX2012’ and consists of a subset of the SALAMI (‘Structural Analysis of Large Amounts of Music Information’) database (Smith, Burgoyne, Fujinaga, De Roure, & Downie , 2011)^10^.

By capitalizing on double-keyed data (two individual annotations present per music piece), the authors computed inter-rater agreement using the FPC-F measure. The authors stated that, while ‘algorithmic segmentations seem to perform similarly to each other, automatic segmentation has not reached human performance’, concluding that, by the year 2011, ‘the state of automatic segmentation is relatively immature’. A meta-analysis of the MIREX structure segmentation task conducted in 2012 was published as Smith and Chew (2013). The study compared evaluation metrics by correlation to find out which ones actually measure different qualities. An attempt was also made to identify the music pieces in the (principally anonymous) MIREX MSS data sets, potentially enabling a more directed analysis of algorithm results. Baselines and inter-rater agreement (‘upper bounds’) for segment boundary recognition have also been reported in Serrà, Müller, Grosche, and Arcos (2014) on the Beatles albums and the RWC-POP data set (see Section 3.2).

The ambiguity of annotating boundaries at a certain level of detail has been demonstrated in Grill and Schlüter (2015): even if annotators agree on the positions of boundaries, they often disagree regarding their assignment to a certain structural level. The authors use the available two-level annotation to let their algorithm learn the criteria for a boundary to belong to one of the two structural levels, thereby also raising the overall segmentation quality. Recently, a framework for the hierarchical evaluation of segment boundary detection, acknowledging the non-binary nature of boundary perception has been presented in McFee, Nieto, and Bello (2015).

## 3. Data

### 3.1. Audio music similarity

For our meta analysis of audio music similarity (AMS) we use the data from the ‘Audio Music Similarity and Retrieval’ tasks from 2006 to 2014^11^ within the annual MIREX (Downie , 2006) evaluation campaign for MIR algorithms. The AMS 2006 data will be used to derive an upper performance bound which will then be compared to results from AMS 2007 to 2014 in Section 4.

For the AMS 2006 task, 5000 songs were chosen from the so-called ‘uspop’, ‘uscrap’ and ‘cover song’ collections. Each of the participating six systems then returned a 5000
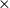
5000 AMS distance matrix. From the complete set of 5000 songs, 60 songs were randomly selected as queries and the first five most highly ranked songs out of the 5000 were extracted for each query and each of the six systems (according to the respective distance matrices). These five most highly ranked songs were always obtained after filtering out the query itself, results from the same artist (i.e. a so-called artist filter was employed (Flexer & Schnitzer , 2010)) and members of the cover song collection (since this was essentially a separate task run together with the AMS task). The distribution for the 60 chosen random songs is highly skewed towards rock music: 22 ROCK songs, 6 JAZZ, 6 RAP&HIPHOP, 5 ELECTRONICA&DANCE, 5 R&B, 4 REGGAE, 4 COUNTRY, 4 LATIN, 4 NEWAGE. Unfortunately the distribution of genres across the 5000 songs is not available, but there is some information concerning the ‘excessively skewed distribution of examples in the database (roughly 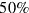 of examples are labelled as Rock/Pop, while a further 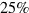 are Rap & Hip-Hop)’^12^. For each query song, the returned results (candidates) from all participating systems were evaluated by human graders. For each individual query/candidate pair, three different human graders provided both a FINE score (from 0 (failure) to 10 (perfection)) and a BROAD score (not similar, somewhat similar, very similar) indicating how similar the songs are in their opinion. This altogether gives 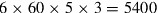 human FINE and BROAD gradings. Please note that since some of the query/candidate pairs are identical for some algorithms (i.e. different algorithms returned identical candidates) and since such identical pairs were not graded repeatedly, the actual number of different FINE and BROAD gradings is somewhat smaller.

Starting with the AMS task 2007, a number of small changes to the overall procedure was introduced. Each participating algorithm was given 7000 songs chosen from the ‘uspop’, ‘uscrap’ and ‘american classical’ and ‘sundry’ collections. Therefore there is only a partial overlap in music collections (‘uspop’ and ‘uscrap’) compared to AMS 2006. Also from then on 30 s clips instead of the full songs were being used both as input to the algorithms and as listening material for the human graders. For the subjective evaluation of music similarity, 100 query songs were randomly chosen representing the 10 genres found in the database (i.e. 10 queries per genre). The whole database consists of songs from equally sized genre groups: BAROQUE, COUNTRY, EDANCE, JAZZ, METAL, RAPHIPHOP, ROCKROLL, ROMANTIC, BLUES, CLASSICAL. Therefore there is only a partial overlap of genres compared to AMS 2006 (COUNTRY, EDANCE, JAZZ, RAPHIPHOP, ROCKROLL). As with AMS 2006, the five most highly ranked songs were then returned per query as candidates (after filtering for the query song and songs from the same artist). For AMS tasks 2012, 2013 and 2014, 50 instead of 100 query songs were chosen and 10 instead of five most highly ranked songs returned as candidates.

Probably the one most important change to the AMS 2006 task is the fact that starting from 2007, every query/candidate pair was only being evaluated by a single user. Therefore the degree of inter-rater agreement cannot be analysed anymore. For every AMS task, the subjective evaluation therefore results in 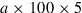 human FINE and BROAD gradings, with *a* being the number of participating algorithms, 100 the number of query songs and 5 the number of candidate songs. For AMS 2012, 2013 and 2014 this changed to 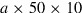, which yields the same overall number. These changes are documented on the respective MIREX websites, but also in a MIREX review article covering all tasks of the campaign (Downie, Ehmann, Bay, & Jones , 2010). For AMS 2007 and 2009, the FINE scores range from 0 to 10, from AMS 2010 onwards from 0 to 100. There was no AMS task in MIREX 2008.

### 3.2. Music segmentation

The SALAMI database, as partly used in MIREX, contains over 1500 annotations of musical recordings from different genres and origins, including music by the Beatles and parts of RWC. With the latest SALAMI version 2.0, the annotations of a total of 1164 recordings (with 763 double-annotated) are publicly available. This data set has been produced on the fundaments of a detailed annotator’s guide,^13^ describing the specifications and nomenclature of structural annotation within the SALAMI framework. This guide ensures that all annotations, especially two annotation versions of the same music piece, follow the same fundamentals (e.g. level of detail), making them reproducible and comparable in the first place. Notably, SALAMI offers annotations on two levels of detail—‘large scale’ or ‘functional’, and ‘small scale’—incorporating the hierarchical nature of annotation. Currently, within the MIREX framework, only the ‘large scale’ annotations are used. For our evaluation purposes in this contribution, we focus on the SALAMI-based ‘MIREX2012’ data set of MIREX, because of the perfect comparability of multiple annotations as a requirement for reliable analysis of inter-rater agreement.

The results of all algorithms participating in MIREX have been made public^14^, along with the respective ground-truth used for the evaluation. By matching the MIREX ground-truth data to the publicly available SALAMI annotations, we have been able to identify the origins of 756 music pieces, 676 of which are double-annotated. For all the matched music pieces we also have available the generated annotation results of nine algorithms (abbreviated KSP1, KSP2, KSP3, MHRAF1, OYZS1, SBV1, SMGA1, SMGA2, and SP1) of the year 2012, eight algorithms (CF5, CF6, MP1, MP2, RBH1, RBH2, RBH3, RBH4) of 2013, six algorithms (NB1, NB2, NB3, NJ1, SUG1, SUG2) of 2014, and four algorithms (GS1, GS3, CC1, MC1) of 2015. The descriptions for each of these algorithms are available from the MSS MIREX web site.

Evaluation within the MIREX campaign is performed using the NEMA (‘Networked Environment for Music Analysis’) framework^15^ which covers all possible MIREX tasks. For the MSS task, we only look at the evaluation measures for ‘segment boundary recovery’ (SBR), the most widely used measure in the literature. SBR refers to the question whether a predicted boundary falls into a temporal window around a ground-truth boundary. In the existing literature, as well as in the MIREX MSS task, two window sizes (or, tolerances) are commonly used: 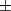
0.5 and 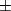
3 s. For each file in the data set, precision and recall rates, as well as the composite 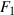 score are computed. The quality of an algorithm is then characterized by these values averaged over all the files in the data set. Usually, the 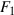 score is used to rank the algorithms. It is by far the most common measure used in the literature. Nieto, Farbood, Jehan, and Bello (2014) have identified a 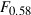 measure to be more perceptually informative than 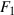, but this is a relatively new and not well established finding. For our limited purposes, handling the immense NEMA framework would have been too time-consuming. We have therefore chosen to resort to the newer, slim ‘mir_eval’ package (Raffel et al. , 2014). Differences in the resulting scores may arise due to mir_eval using a refined maximum bipartite matching algorithm, instead of the greedy matching strategy used in NEMA. Apart from that, we have normalized the annotations by stripping off silent leading and trailing segments prior to performing the evaluation.

## 4. Results

We will first give results for audio music similarity and next for structure segmentation. This corresponds to music similarity between and within music, respectively.

### 4.1. Audio music similarity

In our meta analysis of the AMS tasks from years 2006 to 2014, we will focus on the FINE scores of the subjective evaluation conducted by the human graders. The reason is that the FINE scores provide more information than the BROAD scores which only allow for three categorical values. It has also been customary for the presentation of AMS results to mainly compare average FINE scores for the participating algorithms.

#### 4.1.1. Analysis of inter-rater agreement

Our first analysis is concerned with the degree of inter-rater agreement achieved in the AMS task 2006, which is the only year every query/candidate pair has been evaluated by three different human graders. Previous analysis of AMS results has concentrated on BROAD scores and used Fleiss’ Kappa as a measure of agreement (see Section 2). Since the Kappa measure is only defined for the categorical scale, we use the Pearson correlation 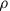 between FINE scores of pairs of graders. The average correlation is 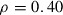. Taking the square of the observed value of 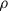, we can see that only 16% of the variance of FINE scores observed in one grader can be explained by the values observed for the respective other grader (see, e.g. Cohen (1988) on 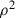 measures). Therefore, this is the first indication that agreement between raters in the AMS task is rather low.

**Figure 1.**
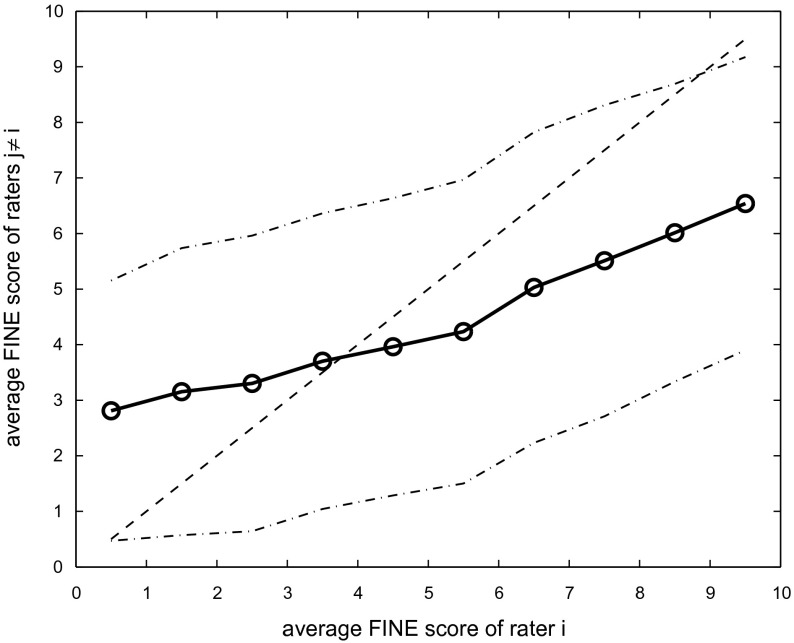
Average FINE score inter-rater agreement for different intervals of FINE scores (solid line) 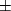 one standard deviation (dash-dot lines). Dashed line indicates theoretical perfect agreement.

Next we plotted the average FINE score of a rater *i* for all query/candidate pairs, which he or she rated within a certain interval of FINE scores *v*, versus the average FINE scores achieved by the other two raters 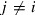 for the same query/candidate pairs. We therefore explore how human graders rate pairs of songs which another human grader rated at a specific level of similarity. The average results across all raters and for intervals *v* ranging from 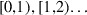 to 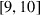 are plotted in Figure 1. It is evident that there is a considerable deviation from the theoretical perfect agreement which is indicated as a dashed line. Pairs of query/candidate songs which are rated as being very similar (FINE score between 9 and 10) by one grader are on average only rated at around 6.5 by the two other raters. On the other end of the spectrum, query/candidate pairs rated as being not similar at all (FINE score between 0 and 1) receive average FINE scores of almost 3 by the respective other raters.

Looking at the standard deviation around the averages plotted as dash-dotted lines, it is also evident that there is a considerable variation of FINE scores within the data. It is also interesting to note, that the distribution of query/candidate pairs across intervals is far from even. Whereas 2004 query/candidate pairs fall into the 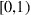 interval, this decreases monotonically to 333 for the 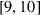 interval. This is also reflected in the fact that the average across all FINE scores of all raters is only 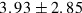 (mean 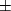 standard deviation).

Returning to ratings within the highest interval, one can see that query/candidate pairs that have been rated between 9 and 10 by one grader have received an average rating of 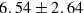 by the respective other two graders. This constitutes an upper bound 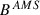 for the average FINE scores of the AMS task 2006. This upper bound is the maximum of average FINE scores that can be achieved within such an evaluation setting. This upper bound is due to the fact that there is a considerable lack of agreement between human graders. What sounds very similar to one of the graders will on average not receive equally high scores by other graders.

As has been explained in Section 3.1, the data used for AMS 2006 differs in two major aspects from data used for later renditions of the AMS task. It is heavily skewed towards genres ROCK (50% of all songs) and RAP&HIPHOP (25%). There are also only five genres that are overlapping between AMS 2006 and AMS 2007–2014: COUNTRY, EDANCE, JAZZ, RAPHIPHOP, ROCKROLL. Although the whole evaluation protocol in all AMS tasks over the years is almost identical, it is nevertheless debatable how strictly the upper bound 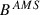 from AMS 2006 applies to the AMS results of later years. We therefore derive two more upper bounds which take these problems into account.

The upper bound 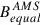 weighs each of the nine genres from AMS 2006 equally by randomly choosing an equal amount of query songs from each genre. The full 60 query songs are comprised of 22 ROCK songs, 6 JAZZ, 6 RAP&HIPHOP, 5 ELECTRONICA&DANCE, 5 R&B, 4 REGGAE, 4 COUNTRY, 4 LATIN, 4 NEWAGE. The minimum number of query songs per genre is four. We therefore choose four query songs from each genre which leaves us with 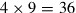 query songs and 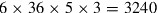 FINE scores. Just as for 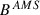, we select pairs of scores where at least one of the scores is in the interval 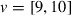 and compute the average pairwise inter-rater agreement. This randomization procedure is done ten times and the average inter-rater agreement closest to the mean performance of the ten trials is kept as the upper bound 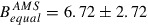, which is based on 212 pairs of scores. We chose the average inter-rater agreement closest to the mean in order to counter effects of the random samples drawn from all query songs.

The upper bound 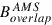 is based on randomly choosing an equal amount of query songs only from the five genres which are overlapping between AMS 2006 and AMS 2007–2014. Choosing four songs from each of the five genres leaves us with 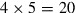 query songs and 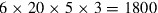 FINE scores. Again we select pairs of scores where at least one of the scores is in the interval 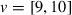 and compute the average pairwise inter-rater agreement. This randomization procedure is done ten times and again the average inter-rater agreement closest to the mean performance of the ten trials is kept as the upper bound 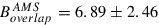, which is based on 143 pairs of scores.

Both alternative upper bounds are higher than 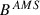 with 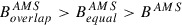. This means that taking into account the skewness of the genre distribution as well as including only genres that overlap with later AMS renditions yields slightly higher upper bounds. The reason is that average FINE scores per genre are different for individual genres, with e.g. ROCK having an average FINE score of 3.88 versus all genres together having an average of 3.93. Of course the most correct approach would be to compute upper bounds for every AMS year separately based on FINE scores from the respective years, but this is impossible since unfortunately multiple annotations only exist for AMS 2006. By computing three different upper bounds based on AMS 2006 we tried to make the most of the inter-rater data that is available.

#### 4.1.2. Comparison to the upper bound

We will now compare the performance of the respective best participating systems in AMS 2006, 2007, and 2009 to 2014 to the upper bounds of average FINE scores we have retrieved in Section 4.1.1. These upper bounds, that can possibly be achieved due to the low inter-rater agreement, result from the analysis of the AMS 2006 task. To make FINE scores comparable over the years, we converted all scores from 2006 to 2009, as well as the upper bounds, to the 0 to 100 range via a multiplication by 10.

**Figure 2.**
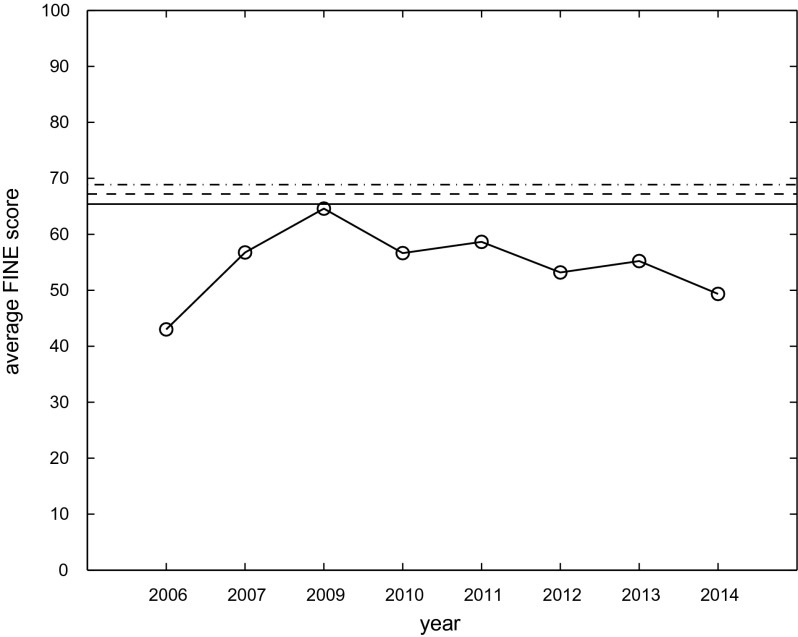
Average FINE score of best performing system (*y*-axis) versus year (*x*-axis) plotted as circles connected via thick solid line. Upper bounds 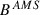 (solid), 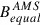 (dashed) and 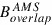 (dash-dot) plotted as horizontal lines.

In Figure 2 we have plotted the average FINE score of the highest performing participants of AMS tasks 2006, 2007, and 2009 to 2014. These highest performing participants are the ones that achieved the highest average FINE scores in the respective years. In terms of statistical significance, the performance of these top algorithms is often at the same level as a number of other systems. We have also plotted the three upper bounds 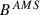, 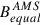 and 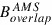. As can be seen the performance peaked in the year 2009 with average FINE scores in all subsequent years always being a little lower. In Table 1 we show the results of a series of *t*-tests^16^ comparing the performance to the three upper bounds. Table 1 gives the AMS year, the abbreviated name of the winning entry, the mean performance, its standard deviation and the resulting *t*-values. The degrees of freedom (*df*) for AMS 2006 are 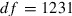 (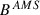), 1110 (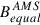) and 1041 (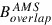). For all other AMS years we have 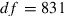 (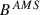), 710 (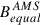) and 641 (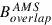). The level of confidence is always 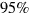 and the critical value for all *t*-tests is 1.96. Only the best entry from year 2009 (PS2) reaches the performance of all three upper bounds, the best entries from all other years are statistically significant below all of the upper bounds.

**Table 1.** Comparison of best system versus three upper bounds 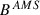, 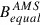 and 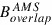 due to low inter-rater agreement. Mean FINE scores plus standard deviations and *t*-test statistics are shown. Differences that are statistically **not** significant are given in bold.

year	system	mean FINE s	t()	t()	t()
2006	EP	43.01 29.80	12.0612	10.7960	9.8555
2007	PS	56.75 29.12	4.3475	4.4538	4.5329
2009	PS2	64.58 25.17	**0.4415**	**1.2313**	**1.8051**
2010	SSPK2	56.64 26.96	4.6230	4.7569	4.8738
2011	SSPK2	58.64 26.23	3.6248	3.9285	4.1676
2012	SSKS2	53.19 27.99	6.3018	6.1502	6.0626
2013	SS2	55.21 26.31	5.4604	5.4951	5.5525
2014	SS2	49.35 26.37	8.4371	8.0005	7.7289

Interestingly, this system PS2 which gave the peak performance of all AMS years has also participated in 2010 to 2014.^17^ In terms of statistical significance (as measured via Friedman tests as part of the MIREX evaluation), PS2 has performed on the same level with the top systems of all following years. The system PS2 has been submitted by Tim Pohle and Dominik Schnitzer and essentially consists of a timbre and a rhythm component (Pohle, Schnitzer, Schedl, Knees, & Widmer , 2009). Its main ingredients are MFCCs modelled via single Gaussians and Fluctuation patterns. It also uses the so-called P-norm normalization of distance spaces for combination of timbre and rhythm and to reduce the effect of hubness (abnormal behaviour of distance spaces due to high dimensionality, see Flexer, Schnitzer, and Schlüter (2012) for a discussion related to the AMS task and Schnitzer, Flexer, Schedl, and Widmer (2012) on re-scaling of distance spaces to avoid these effects).

As outlined in Section 3, from 2007 on the same database of songs was used for the AMS tasks. However, each year a different set of 100 or 50 songs was chosen for the human listening tests. This fact can explain that the one algorithm participating from 2009 to 2014 did not always perform at the exact same level. After all, not only the choice of different human graders is a source of variance in the obtained FINE scores, but also the choice of different song material. It is noticeable that the performance over the years seems to degrade, with 2014 showing the lowest result so far. One explanation could be that the MIR community, which participates in the MIREX evaluation, has become more critical over the years. Nevertheless, the fact that the one algorithm that reached the upper bounds has so far not been outperformed adds additional evidence that the upper bounds that we obtained indeed are valid.

Another way to look at the performance of PS2 over the years from 2009 to 2014 is to pool all the FINE scores available, since the system PS2 and the database of songs remained the same for all these AMS years. In hindsight, it is therefore possible to treat all six experiments as one big experiment. The average FINE score across AMS 2009 to 2014 achieved by PS2 is 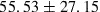, which is 9.05 percentage points lower than its peak performance of 64.58 in 2009. The difference between the FINE scores achieved by the system PS2 and the upper bound 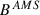 is now significant according to a *t*-test: 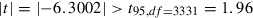. The same is true for 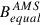 (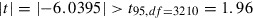) and 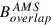 (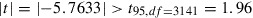). From this retrospective view it seems that the upper bounds were not yet reached and that the peak performance in 2009 might be an outlier due to variance of human graders and/or song material. But such a joint retrospective analysis is of course only possible for systems that participated over many years. It also does not change the fact that with the given AMS test protocol of obtaining only 500 human scores for each participating system based on 100 or 50 query songs, the upper bounds are already within the range of variation of the peak performing systems.

### 4.2. Music segmentation

In our meta analysis of the MSS task, we will first derive upper and lower bounds and then compare them to the top performing MSS algorithms from the years 2012 to 2015.

#### 4.2.1. Analysis of lower and upper bounds

**Figure 3.**
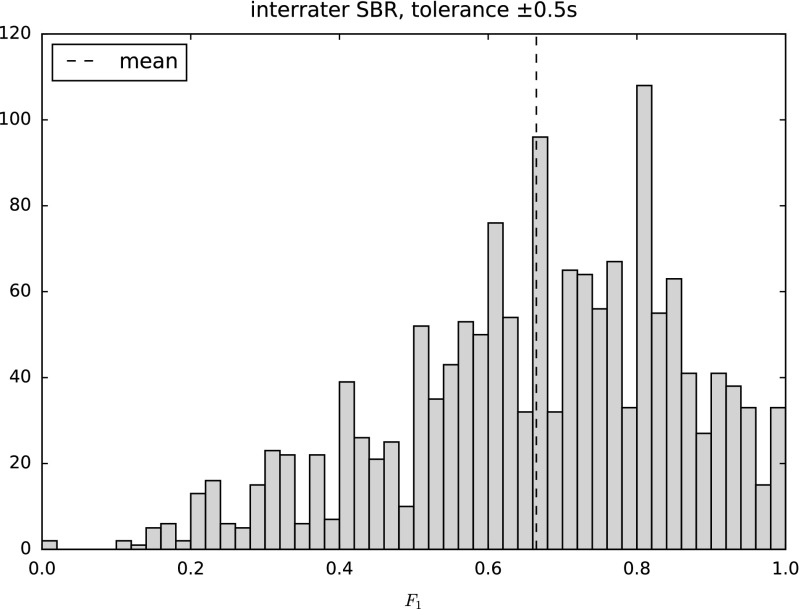
Inter-rater 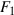 scores plotted as a histogram over all double-annotated pieces contained in the SALAMI data set for a tolerance of 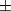
0.5 s. Mean 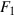 value plotted as a dashed line.

Similar to the AMS task, the algorithms’ resulting scores are bounded by upper limits predetermined by the level of agreement among human annotators. If an algorithm reaches this level (i.e. mean 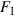 is not different given a certain level of significance), it can be considered as ‘perfect’. Inter-rater agreement between two annotators can be evaluated by taking one annotation as the prediction, and the other one as ground-truth, and vice versa. Figure 3 shows the resulting 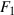 scores for all of the double-annotated music pieces contained in the SALAMI data set, plotted in a histogram. Averaging the 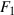 values over all the music pieces yields an estimate for the upper bound 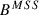. Although there is a high variance on the agreement over the set of music pieces, the mean is well defined owing to the large number of samples.

**Table 2.** 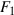
 measures (mean and standard deviation) for lower (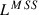) and upper (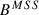) bounds within the SALAMI data set.

tolerance		
0.5 s		
1 s		
3 s		

Additionally, we can calculate a lower bound 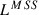 for the SBR evaluation which is the resulting 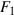 value for guessed (randomly or evenly spaced) boundaries. Table 2 presents lower and upper bounds for the SALAMI data set. For tolerances of 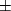
0.5 and 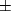
1 s the lower bound is defined by the fact that most of the music pieces have a boundary at the beginning. Using a very wide regular grid, actually representing only the boundary at time 0, the recall rate is close to 1, and precision becomes the inverse of the number of boundaries present in the ground-truth, which turns out to be small for manypieces. It is debatable whether such ‘trivial boundaries’ at the beginning and end of pieces should be considered in the evaluation at all—it is done so in MIREX though. For the widely used tolerance of 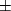
3 s, the highest value of 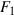 for naïve guessing is achieved at a regular grid width of 6 s, resulting in a remarkably high score of 0.386. It has been found earlier Smith & Chew , 2013) that the 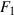 measures for boundary recovery with tolerances of 0.5 and 3 s, respectively, are only weakly correlated, suggesting that ‘locating boundaries to within 3 s and to within 0.5 s are perhaps two distinct skills’. The explorable space for algorithms between lower and upper bounds is greatest for 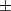
1 s. Unfortunately, this tolerance has not yet been commonly used in the literature, hence we focus mainly on 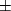
0.5 .

**Table 3.** 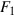
 measures (mean and standard deviation) for lower (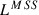) and upper (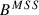) bounds within different genre classes of the SALAMI data set (tolerance is 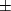
0.5 s).

genre class		
popular		
jazz		
classical		
world		

Table 3 lists lower and upper bounds at a tolerance of 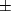
0.5 s for various genre classes of the SALAMI data set. The difference in inter-rater agreement between the genres ‘popular’ and ‘jazz’ on the one hand (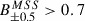), and the structurally more complex genres ‘classical’ and ‘world’ (with 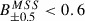) becomes obvious. This same separation is still present for a loosened temporal tolerance of 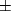
3 s, where the exact precision of segment positions (obviously a difficult issue in classical music) is less important.

#### 4.2.2. Comparison to the upper bound

Tables 4 and 5 show comparisons of the best algorithms for the MIREX MSS task conducted in the years 2012 through 2015 with the upper bound 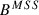 for tolerances of 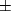
0.5 s and 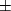
3 s, respectively. The boundary retrieval 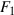 measures calculated on the algorithms’ predicted boundaries lie consistently and significantly below the values computed on the basis of inter-rater agreement (given in Table 2). Although the results have consistently improved over the years 2012 to 2015, the difference of the mean 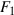 value for the best algorithm GS3 (for a tolerance of 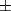
0.5 s) to the upper bound is still highly significant according to a paired *t*-test: 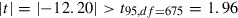 (confidence level of 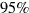, 675 degrees of freedom). The same applies for best-performing GS3 at a tolerance 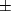
3 s, with 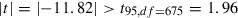.

**Table 4.** Comparison of best algorithm per MIREX edition on the SALAMI data set versus upper bound 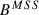 for a tolerance of 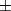
0.5 s. Boundary recognition 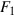 mean values and standard deviations, and paired *t*-test statistics are shown.

year	algorithm	(for tol. 0.5 s)	
2012	KSP2		
2013	MP2		
2014	SUG1		
2015	GS3		

**Table 5.** Comparison of best algorithm per MIREX edition on the SALAMI data set versus upper bound 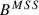 for a tolerance of 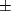
3 s. Boundary recognition 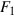 mean values and standard deviations, and paired *t*-test statistics are shown.

year	algorithm	(for tol. 3 s)	
2012	KSP3		
2013	MP2		
2014	SUG2		
2015	GS3		

Analysing the algorithms’ performance for each genre separately, results comparable to the human agreement levels are achieved by GS3 at a tolerance of 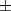
0.5 s. For the genres of world music (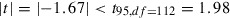) and classical music (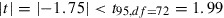), where the upper bounds are at comparably low levels, the differences are not significant anymore.

The results show that there still is some headroom for improvements of algorithmic approaches to the problem of structural segmentation in music compared to human annotation. For genres where human annotators disagree to a greater extent, i.e. classical and world music, the results of state-of-the-art MSS algorithms are no longer significantly worse than human annotation. Note that in Table 5 SUG2 and GS3 evaluate to the same average 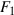 score, but that the paired *t*-values are very different. GS3 performs much better than SUG2 relative to the upper bounds given by inter-rater agreement. Apart from that, the evaluation conducted in the MIREX MSS task is incomplete because, at the moment, it completely neglects the inherent hierarchical nature of structural analysis.

## 5. Discussion

Our meta analysis of editions of the MIREX ‘Audio Music Similarity and Retrieval’ (AMS) and ‘Music Structural Segmentation’ (MSS) tasks has produced somewhat sobering results. Due to limited inter-rater agreement there exist upper bounds of performance in subjective evaluation of the respective music similarity tasks. Such upper bounds of agreement will always exist when a number of different people deal with concepts as complex as those of music similarity and segmentation. Whereas the upper bound for the AMS task has very likely already been reached, there still is some room for improvement in the MSS task. In discussing our results we want to give a number of recommendations on how MIR research could deal with the problem of limited inter-rater agreement.

**Ask more specific questions.** The fact that in the MIREX AMS task the notion of similarity between pieces of music is not defined very clearly lies at the heart of the problem. After all, to ‘sound similar’ does mean something quite different to different people listening to diverse music. As a consequence, an algorithm that has reached this upper bound of performance already in 2009 has not been outperformed ever since. Following our argumentation, this algorithm cannot be outperformed since any additional performance will be lost in the variance of the different human graders. As for the MSS task, the definition of similarity within pieces of music seems to have been formulated a lot clearer and resulted in a detailed annotator’s guide. This much clearer evaluation goal and the corresponding higher grader agreement might be part of an explanation why the upper bound in MSS has not yet been reached.

In order to ask a more specific evaluation question for the AMS task, it is probably necessary to research what the concept of music similarity actually means to human listeners. Such an exploration of what perceptual qualities are relevant to human listeners has already been conducted in the MIR community for the specific case of textural sounds (Grill, Flexer, & Cunningham , 2011). Textural sounds are sound snippets that appear stationary as opposed to evolving over time and are therefore much simpler and constrained than entire music pieces. By conducting mixed qualitative-quantitative interviews the authors were able to show that qualities like ‘high–low’, ‘smooth–coarse’ or ‘tonal–noisy’ are important to humans discerning textural sounds. A similar approach could be explored for real song material, probably starting with a limited subset of genres. After such perceptual qualities have then been identified, future AMS tasks could ask human graders how similar pairs of songs are according to a specific quality of the music. Such qualities might not necessarily be straightforward musical concepts like melody, rhythm, or tempo, but rather more abstract notions like instrumentation, genre or specific production effects signifying a certain style. Such a more fine-grained approach to music similarity would hopefully raise inter-rater agreement and make more room for improvements in modelling music similarity.

**Care about confounding variables.** It has been noted before (Schedl et al. , 2013) that, of course, ‘[...] the basicstructure of MIR experiments is the same as in any other experimental situation: the objective is to measure the effect of different treatments on a dependent variable’. In the case of AMS and MSS the treatments are the different algorithms that model music similarity or estimate segment boundaries. The dependent variables are the FINE scores for AMS and the 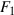 results for MSS, but there are many other factors that are able to influence the results of the algorithms in the AMS and MSS tasks. For the AMS task, obvious examples for confounding variables are the level of expertise of the human graders or their familiarity with the music pieces that are part of the evaluation. One specific example we found in our analysis of the MSS task is the influence of the different genre classes that are part of the database. We have found that the agreement between human annotators varies widely over the spectrum of contained music pieces, involving a notable correlation with specific genre classes. Evidently, the level of agreement is a function of the complexity of the task, with pop music being composed of a generally more distinct and unambiguous structure than, e.g. classical music (see Section 4.2.1). Any factor that is able to influence the relation between treatments and dependent variables are confounding variables that need to be controlled by making them part of the experimental design. This can either be done by narrowing the research question to a more controlled group of human subjects (e.g. only those familiar with classical music) and music material (e.g. classical music only). This of course means asking research questions of a much more limited scope and smaller focus. The other option is to record all these control variables (e.g. familiarity with musical genre yes/no, type of music genre used in evaluation) and analyse their influence on the relation of treatments and dependent variables. To a certain extent this could entail a combinatorial explosion of control variables, which might render it intractable to control for everything that might influence the human perception of music similarity.

**Evaluate complete MIR systems in a holistic way.** Last but not least it has been noted repeatedly that evaluation of abstract music similarity detached from a specific user scenario and corresponding user needs might not be meaningful at all (Hu & Liu , 2010; Lee & Cunningham , 2013; Schedl et al. ,^2013^) Instead, the MIR community might have to change to evaluation of complete music retrieval systems, thereby opening a whole new chapter for MIR research. Such an evaluation of a complete real life MIR system could centre around a specific task for the users (e.g. building a playlist or finding specific music), thereby making the goal of the evaluation much clearer. This has already been named as one of the grand challenges for future MIR research (Serra et al. , 2013). Such a user centred evaluation has already happened at the tenth MIREX anniversary in 2014: the ‘MIREX Grand Challenge 2014: User Experience (GC14UX)’.^18^ The task for participating teams was to create a web-based interface that supports users looking for background music for a short video. Systems were rated by human evaluators on a number of important criteria with respect to user experience. However, a first analysis (Lee, Hu, Choi, & Downie , 2015) of the results showed that it is very hard to find statistically significant differences between the three participating systems. It seems that the differences in user interfaces of the three systems are quite important for the evaluators and might blur differences in models of music similarity at the core of the complete MIR systems. Therefore it is still unclear how fruitful such a holistic evaluation can be.

In finishing this section on recommendations concerning the problem of limited inter-rater agreement, we would like to again stress that only future research will be able to show the usefulness and feasibility of the above outlined suggestions. At the moment it is still an open research question what more specific questions concerning music similarity are, whether control of confounding variables will be feasible and how this can be combined with more holistic approaches towards MIR evaluation. It could very well be that future MIR evaluation campaigns will have to ‘refocus on a core set of better-defined tasks, of a lower-level, more likely to generate insights about human perception of music’ (Aucouturier & Bigand , 2013). As has already been demanded (Aucouturier & Bigand , 2013), this will make a temporary moratorium of higher-level tasks like AMS necessary. Incidently, there was no MIREX AMS task in 2015, since only our own research team, again sending the same peak performing system PS2 for the seventh year, wanted to participate.

## 6. Conclusion

In our paper we have raised the important issue of the limited inter-rater agreement in human evaluation of music information retrieval systems. Since human appraisal of phenomena as complex and multi-dimensional as music similarity is highly subjective and depends on many factors such as personal preferences and past experiences, evaluation based on human judgments naturally shows high variance across subjects. This lack of inter-rater agreement presents a natural upper bound for performance of automatic analysis systems. We have demonstrated and analysed this problem in the context of the MIREX ‘Audio Music Similarity and Retrieval’ and ‘Structural Segmentation’ tasks. For the ‘Audio Music Similarity and Retrieval’ task, the upper bound has seemingly already been reached in 2009 and not been surpassed since then, thereby preventing any further progress in this direction. For the ‘Structural Segmentation’ task, the upper bound is already within reach for some specific types of music.

Our work has also made it clear that any evaluation of MIR systems, that is based on ground truth annotated by humans, has the same fundamental problem. Other examples from the MIREX campaign include such diverse tasks as ‘Symbolic Melodic Similarity’ or ‘Audio Classification’, which are all based on human annotations with varying degrees of ambiguity. Future research should explore upper bounds of performance for these many other MIR tasks based on human annotated data.

Finally we have also discussed ways to deal with the problem of limited inter-rater agreement, which might make it possible to raise the respective upper bounds achievable by MIR systems.

## Footnotes

1 
http://www.music-ir.org/mirex, accessed 2016-02-25.

2 There was no MIREX AMS task in 2015, see also Section 5.

3 
http://ifs.tuwien.ac.at/mir/audiosegmentation.html#anchor_corpus, accessed 2016-02-25

4 
http://www.cs.tut.fi/sgn/arg/paulus/beatles_sections_TUT.zip, accessed 2016-02-25

5 
http://isophonics.net/content/reference-annotations, accessed 2016-02-25

6 
http://www.icce.rug.nl/~soundscapes/DATABASES/AWP/awp-a.shtml, accessed 2016-02-25

7 
https://staff.aist.go.jp/m.goto/RWC-MDB/AIST-Annotation, accessed 2016-02-25

8 
http://musicdata.gforge.inria.fr/structureAnnotation.html, accessed 2016-02-25

9 
http://www.quaero.org, accessed 2016-02-25

10 
https://ddmal.music.mcgill.ca/research/salami/annotations, accessed 2016-02-25

11 The results and details can be found at: http://www.music-ir.org/mirex/wiki/MIREX_HOME, accessed 2016-02-25

12 This is stated in the 2006 MIREX AMS results: http://www.music-ir.org/mirex/wiki/2006:Audio_Music_Similarity_and_Retrieval_Results, accessed 2016-02-25

13 
http://www.music.mcgill.ca/~jordan/salami/SALAMI-Annotator-Guide.pdf, accessed 2016-02-25

14 e.g. for the SALAMI data set in 2015:http://nema.lis.illinois.edu/nema_out/mirex2015/results/struct/salami/files.html, accessed 2016-02-25

15 
https://code.google.com/p/nemadiy, accessed 2016-02-25

16 For information concerning statistical testing in the context of MIR as well as on *t*-tests see, e.g. (Flexer , 2006).

17 Under the name of PS1 from 2010 onwards.

18 
http://www.music-ir.org/mirex/wiki/2014:GC14UX, accessed 2016-02-25

## References

[CIT0001] Allan H., Müllensiefen D., Wiggins G.A. (2007). Methodological considerations in studies of musical similarity.

[CIT0002] Aucouturier J.J., Minett J.W., Wang W.S.-Y. (2009). Sounds like teen spirit: Computational insights into the grounding of everyday musical terms. *Language, evolution and the brain*.

[CIT0003] Aucouturier J.J., Bigand E. (2013). Seven problems that keep MIR from attracting the interest of cognition and neuroscience. *Journal of Intelligent Information Systems*.

[CIT0004] Bimbot F., Deruty E., Sargent G., Vincent E. (2012). *Methodology and conventions for the latent semiotic annotation of music structure (Technical report: INRIA-IRISA-METISS)*.

[CIT0005] Bruderer M.J., McKinney M., Kohlrausch A. (2006). Structural boundary perception in popular music. *Proceedings of the 7th International Conference on Music Information Retrieval*.

[CIT0006] Cohen J. (1988). *Statistical power analysis for the behavioral sciences*.

[CIT0007] Downie J.S. (2006). The Music Information Retrieval Evaluation eXchange (MIREX). *D-Lib Magazine*.

[CIT0008] Downie J.S., Ehmann A.F., Bay M., Jones M.C. (2010). The music information retrieval evaluation exchange: Some observations and insights. *Advances in music information retrieval (Studies in Computational Intelligence*.

[CIT0009] Downie J.S., Hu X., Lee J.H., Choi K., Cunningham S.J., Hao Y., Bainbridge D. (2014). Ten years of MIREX (Music Information Retrieval Evaluation eXchange): reflections, challenges and opportunities. *Proceedings of the International Society for Music Information Retrieval Conference*.

[CIT0010] Ehmann A.F., Bay M., Downie J.S., Fujinaga I., De Roure D. (2011). Music structure segmentation algorithm evaluation: Expanding on MIREX 2010 analyses and datasets. *Proceedings of the 12th International Society for Music Information Retrieval Conference*.

[CIT0011] Fleiss J.L. (1971). Measuring nominal scale agreement among many raters. *Psychological Bulletin*.

[CIT0012] Flexer A. (2006). Statistical evaluation of music information retrieval experiments. *Journal of New Music Research*.

[CIT0013] Flexer A. (2014). On inter-rater agreement in audio music similarity. *Proceedings of the 15th International Society for Music Information Retrieval Conference*.

[CIT0014] Flexer A., Schnitzer D. (2010). Effects of album and artist filters in audio similarity computed for very large music databases. *Computer Music Journal*.

[CIT0015] Flexer A., Schnitzer D., Schlüter J. (2012). A MIREX meta-analysis of hubness in audio music similarity. *Proceedings of the 13th International Society for Music Information Retrieval Conference*.

[CIT0016] Gjerdingen R.O., Perrott D. (2008). Scanning the dial: The rapid recognition of music genres. *Journal of New Music Research*.

[CIT0017] Goto M. (2004). Development of the RWC music database.

[CIT0018] Goto M. (2006). AIST annotation for the RWC music database. *Proceedings of the 7th International Conference on Music Information Retrieval*.

[CIT0019] Grill T., Flexer A., Cunningham S. (2011). Identification of perceptual qualities in textural sounds using the repertory grid method. *Proceedings of the 6th Audio Mostly Conference*.

[CIT0020] Grill T., Schlüter J. (2015). Music boundary detection using neural networks on combined features and two-level annotations. *Proceedings of the 16th International Society for Music Information Retrieval Conference*.

[CIT0021] Jones M.C., Downie J.S., Ehmann A.F. (2007). Human similarity judgments: Implications for the design of formal evaluations.

[CIT0022] Klien V., Grill T., Flexer A. (2012). On automated annotation of acousmatic music. *Journal of New Music Research*.

[CIT0023] Hu X., Liu J. (2010). Evaluation of music information retrieval: Towards a user-centered approach.

[CIT0024] Landis J.R., Koch G.G. (1977). The measurement of observer agreement for categorical data. *Biometrics*.

[CIT0025] Lee J.H., Cunningham S.J. (2013). Toward an understanding of the history and impact of user studies in music information retrieval. *Journal of Intelligent Information Systems*.

[CIT0026] Lee J.H., Hu X., Choi K., Downie J.S. (2015). MIREX Grand Challenge 2014 on User Experience: Qualitative analysis of user feedback. *Proceedings of the 16th International Society for Music Information Retrieval Conference*.

[CIT0027] Lippens S., Martens J., Mulder T.D., Tzanetakis G. (2004). A comparison of human and automatic musical genre classification.

[CIT0028] McFee B., Nieto O., Bello J.P. (2015). Hierarchical evaluation of segment boundary detection. *Proceedings of the 16th International Society for Music Information Retrieval Conference*.

[CIT0029] Nieto O., Farbood M.M., Jehan T., Bello J.P. (2014). Perceptual analysis of the f-measure for evaluating section boundaries in music. *Proceedings of the 15th International Society for Music Information Retrieval Conference*.

[CIT0030] Novello A., McKinney M.F., Kohlrausch A. (2006). Perceptual evaluation of music similarity. *Proceedings of the 7th International Conference on Music Information Retrieval*.

[CIT0031] Pampalk E. (2006). *Computational models of music similarity and their application to music information retrieval (Doctoral thesis)*.

[CIT0032] Pampalk E., Dixon S., Widmer G. (2004). Exploring music collections by browsing different views. *Computer Music Journal*.

[CIT0033] Paulus J., Müller M., Klapuri A. (2010). Audio-based music structure analysis. *Proceedings of the 11th International Conference on Music Information Retrieval*.

[CIT0034] Pohle T., Schnitzer D., Schedl M., Knees P., Widmer G. (2009). On rhythm and general music similarity. *Proceedings of the 10th International Society for Music Information Retrieval Conference*.

[CIT0035] Raffel C., McFee B., Humphrey E.J., Salamon J., Nieto O., Liang D., Ellis D.P.W. (2014). mir\_eval: A transparent implementation of common MIR metrics.

[CIT0036] Schedl M., Flexer A., Urbano J. (2013). The neglected user in music information retrieval research. *Journal of Intelligent Information Systems*.

[CIT0037] Schnitzer D., Flexer A., Schedl M., Widmer G. (2012). Local and global scaling reduce hubs in space. *Journal of Machine Learning Research*.

[CIT0038] Serra X., Magas M., Benetos E., Chudy M., Dixon S., Flexer A., Gomez E., Gouyon F., Herrera P., Jorda S., Paytuvi O., Peeters G., Schl{\"u}ter J., Vinet H., Widmer G., Peeters G. (2013). *Roadmap for Music Information ReSearch*.

[CIT0039] Serrà J., Müller M., Grosche P., Arcos J.L. (2014). Unsupervised music structure annotation by time series structure features and segment similarity. *IEEE Transactions on Multimedia, Special Issue on Music Data Mining*.

[CIT0040] Seyerlehner K., Widmer G., Knees P. (2010). A comparison of human, automatic and collaborative music genre classification and user centric evaluation of genre classification systems. *Proceedings of the 8th International Workshop on Adaptive Multimedia Retrieval*.

[CIT0041] Smith J.B.L., Burgoyne J.A., Fujinaga I., De Roure D., Downie J.S. (2011). Design and creation of a large-scale database of structural annotations. *Proceedings of the International Society for Music Information Retrieval Conference*.

[CIT0042] Smith J.B.L., Chew E. (2013). A meta-analysis of the MIREX structure segmentation task. *Proceedings of the 14th International Society for Music Information Retrieval Conference*.

[CIT0043] Sturm B.L. (2013). Classification accuracy is not enough. *Journal of Intelligent Information Systems*.

[CIT0044] Urbano J., Downie J.S., McFee B., Schedl M. (2012). How significant is statistically significant? The case of audio music similarity and retrieval. *Proceedings of the 13th International Society for Music Information Retrieval Conference*.

[CIT0045] Vignoli F. (2004). Digital music interaction concepts: A user study. *Proceedings of the 5th International Conference on Music Information Retrieval*.

[CIT0046] West K. (2008). *Novel techniques for audio music classification and search*.

[CIT0047] Wiggins G. (2009). Semantic gap?? Schemantic schmap!! Methodological considerations in the scientific study of music. *Proceedings of the 11th IEEE International Symposium on Multimedia*.

